# Interobserver reliability of the Gehweiler classification and treatment strategies of isolated atlas fractures: an internet-based multicenter survey among spine surgeons

**DOI:** 10.1007/s00068-020-01494-y

**Published:** 2020-09-12

**Authors:** Markus Laubach, Miguel Pishnamaz, Matti Scholz, Ulrich Spiegl, Richard Martin Sellei, Christian Herren, Frank Hildebrand, Philipp Kobbe

**Affiliations:** 1 Department of Orthopaedic Trauma and Reconstructive Surgery, RWTH Aachen University Hospital, Pauwelsstraße 30, 52074 Aachen, Germany; 2 Center for Spinal Surgery and Neurotraumatology, Berufsgenossenschaftliche Unfallklinik Frankfurt, Frankfurt, Germany; 3 Department of Orthopaedics, Trauma Surgery and Reconstructive Surgery, University of Leipzig, Leipzig, Germany; 4 Department of Trauma Surgery and Orthopaedics, Sana Klinikum, Offenbach, Germany

**Keywords:** Interobserver reliability, Atlas fracture, Gehweiler classification, Kappa statistics, Treatment strategy

## Abstract

**Purpose:**

Atlas (C1) fractures are commonly rated according to the Gehweiler classification, but literature on its reliability is scarce. In addition, evaluation of fracture stability and choosing the most appropriate treatment regime for C1-injuries are challenging. This study aimed to investigate the interobserver reliability of the Gehweiler classification and to identify whether evaluation of fracture stability as well as the treatment of C1-fractures are consistent among spine surgeons.

**Methods:**

Computed tomography images of 34 C1-fractures and case-specific information were presented to six experienced spine surgeons. C1-fractures were graded according to the Gehweiler classification, and the suggested treatment regime was recorded in a questionnaire. For data analyses, SPSS was used, and interobserver reliability was calculated using Fleiss’ kappa (*κ*) statistics.

**Results:**

We observed a moderate reliability for the Gehweiler classification (*κ* = 0.50), the evaluation of fracture stability (*κ* = 0.50), and whether a surgical or non-surgical therapy was indicated (*κ* = 0.53). Type 1, 2, 3a, and 5 fractures were rated stable and treated non-surgically. Type 3b fractures were rated unstable in 86.7% of cases and treated by surgery in 90% of cases. Atlas osteosynthesis was most frequently recommended (65.4%). Overall, 25.8% of type 4 fractures were rated unstable, and surgery was favoured in 25.8%.

**Conclusion:**

We found a moderate reliability for the Gehweiler classification and for the evaluation of fracture stability. In particular, diverging treatment strategies for type 3b fractures emphasise the necessity of further clinical and biomechanical investigations to determine the optimal treatment of unstable C1-fractures.

## Introduction

Isolated atlas (C1) fractures account for 2.0‒13.0% of cervical spine injuries and 1.3‒2.0% of all spinal injuries [1, 2]. Individuals in their mid-20s and over 80 years of age are particularly at risk of sustaining C1-fractures, and the mean age of diagnosis in case of trauma is 64 years [3]. While low-energy trauma causes about 50.0% of all cervical fractures [4], atlas fractures are typically seen after an impact to the vertex of the skull [5]. Therefore, most of the C1-fractures occur during falls, motor vehicle accidents, and when diving into shallow water [6]. The craniovertebral junction is especially vulnerable to spinal instability, because the majority of the cervical spine’s axial rotation occurs at the atlantoaxial complex [7], and flexion and extension are greatest at the occiput-atlas joint [8].

In Europe, the Gehweiler classification [9] is widespread and commonly used to grade atlas fractures [10]. Despite the paucity of literature regarding its reliability, it has formed the basis for recent treatment algorithms for the management of atlas fractures [10, 11]. Furthermore, although the incidence of atlas fractures has nearly doubled during the last two decennia [2], there is only a small amount of evidence regarding the most appropriate treatment for such injuries [12]. This may be since upper cervical spine injuries depend greatly on the conjuncture of concomitant bony and ligamentous injuries [13]. Therefore, assessing fracture stability and choosing the most appropriate treatment is complex. An unstable atlas fracture has been defined as a Jefferson fracture with a lesion of the transverse atlantal ligament (TAL) with an incongruence of the atlanto-occipital joint that results in atlanto-occipital instability or displaced atlantoaxial joint facets [3]. It is widely accepted that surgical treatment is indicated for these unstable and displaced atlas fractures [14]. However, there is a paucity of internationally accepted diagnostic and treatment algorithms [12]. In addition, to the best of our knowledge, no studies have systematically investigated the reliability of the Gehweiler classification, which is fundamental to establish a classification-based treatment algorithm.

Based on ratings of experienced spine surgeons, the aims of this study were to evaluate (1) the interobserver reliability of the Gehweiler classification and (2) the C1-fracture stability as well as (3) to assess treatment strategies according to their prior fracture classification.

## Materials and methods

This internet-based, multicenter survey was performed by presenting case-specific information and radiographic data regarding isolated atlas fractures to spine surgeons affiliated with various Spine Centers throughout Germany. For this purpose, a website was created to make the clinical and radiological data, derived from two centers, available to the spine surgeons. The treatment choices of each participating surgeon were documented using an anonymised questionnaire. The study was approved by the local Ethics Committee (EK 221/18).

### Observer recruitment and case-specific information

In accordance with the principle of selective sampling 20 board-certified (German Society for Spine Surgery) orthopaedic and trauma spine surgeons from the Orthopedic and Trauma Departments of six different Level-I Trauma Centers were invited. Six senior spine surgeons, affiliated with four different Orthopedic and Trauma Departments of Level-I Trauma Centers, accepted the invitation. Each observer had at least 8 years of experience in spine surgery and evaluated the data regarding 34 randomly sorted isolated atlas injuries. The supplied clinical data comprised the age, sex, and neurological status of each patient as well as the origin of injury in each case.

### Gehweiler and Dickman classifications

**Fig. 1:**
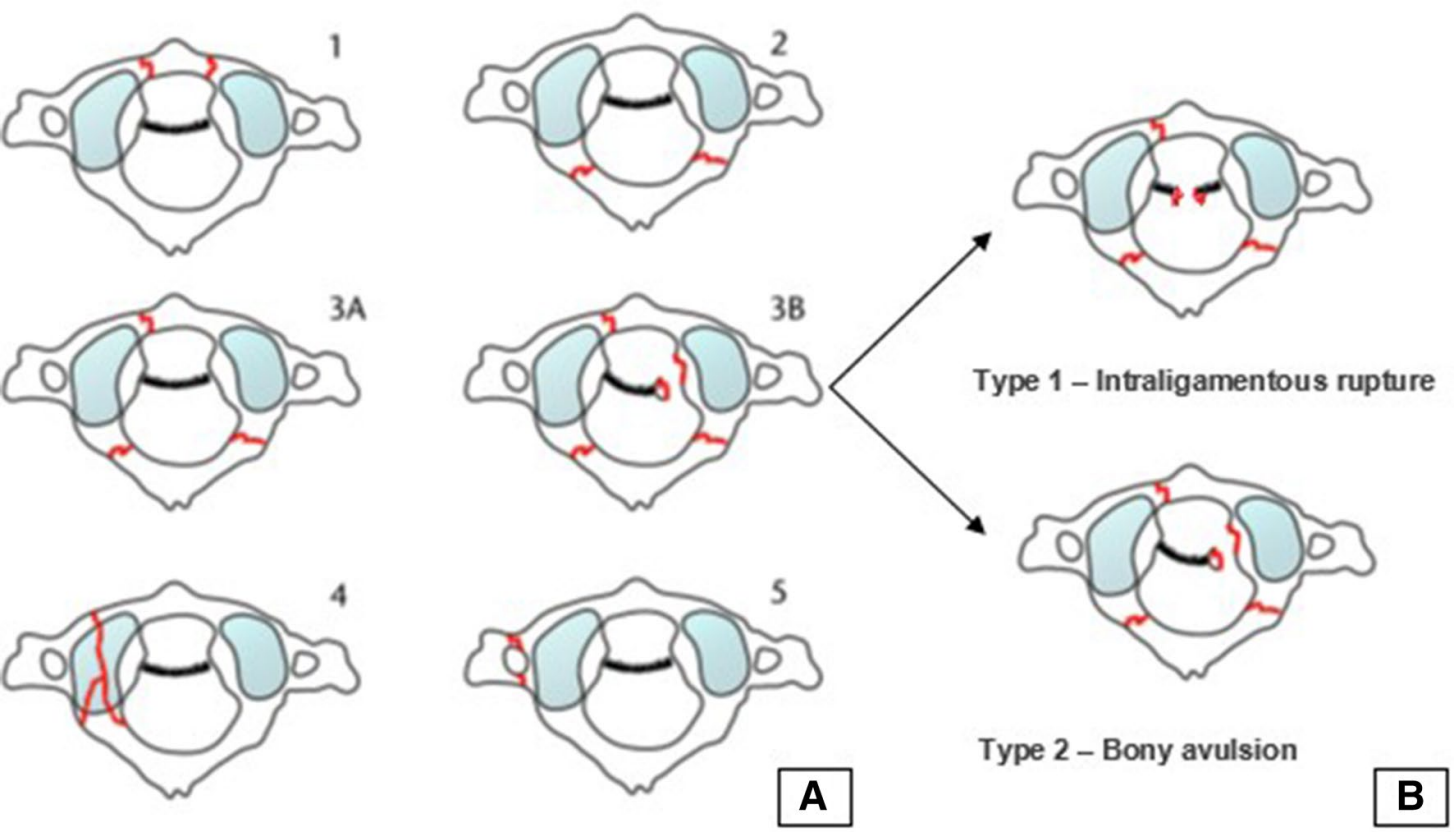
**a** Classification of atlas ring fractures according to Gehweiler [9]; **b** subdivision of Gehweiler type 3b fracture according to Dickman et al. [15]. Adapted from Schleicher et al. [43]

Within a survey, fractures were graded according to the Gehweiler classification. In this classification system, type 1 fractures are those of the anterior arch, type 2 fractures are those of the posterior atlas ring (predominantly bilateral), and type 3 are Jefferson fractures involving the anterior and posterior arch, including types 3a (intact TAL) and 3b, which have either intraligamentous (Dickman type 1) or bony avulsion (Dickman type 2) lesions of the TAL. In addition, type 4 fractures are those of the lateral mass, and type 5 are isolated fractures of the transverse process of the atlas (Fig. 1a, b).

### Study procedures

**Fig. 2:**
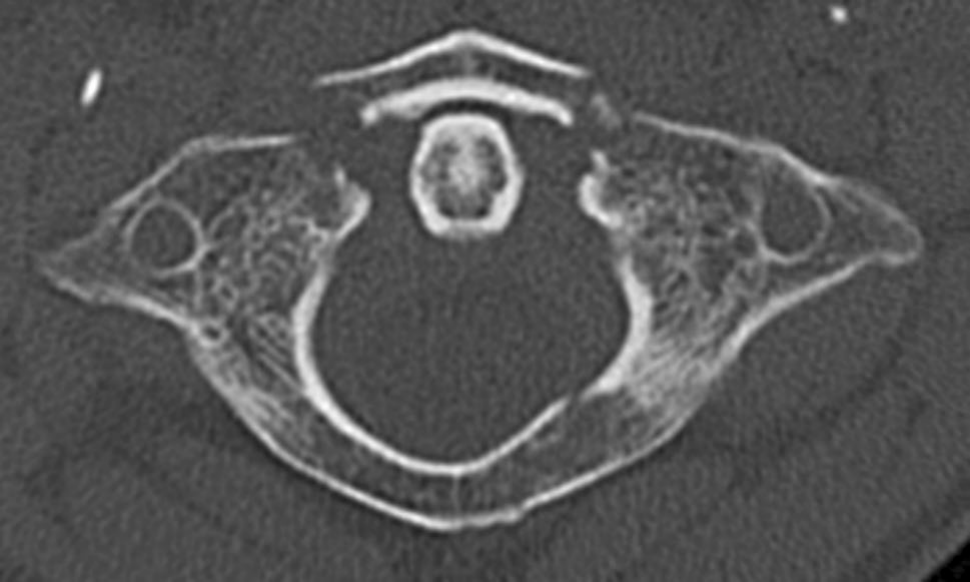
Section of a CT sequence in axial reconstruction of an exemplary case rated by the observers. This case was rated by all observers as a Gehweiler type 3 fracture, but the subclassification into type 3a (50%) and type 3b (50%) was inconsistent and all observers recommended additional magnetic resonance imaging. While four spine surgeons opted for a non-surgical treatment, one observer suggested a Goel–Harms fixation and one observer an isolated atlas osteosynthesis. *CT* computed tomography

**Fig. 3:**
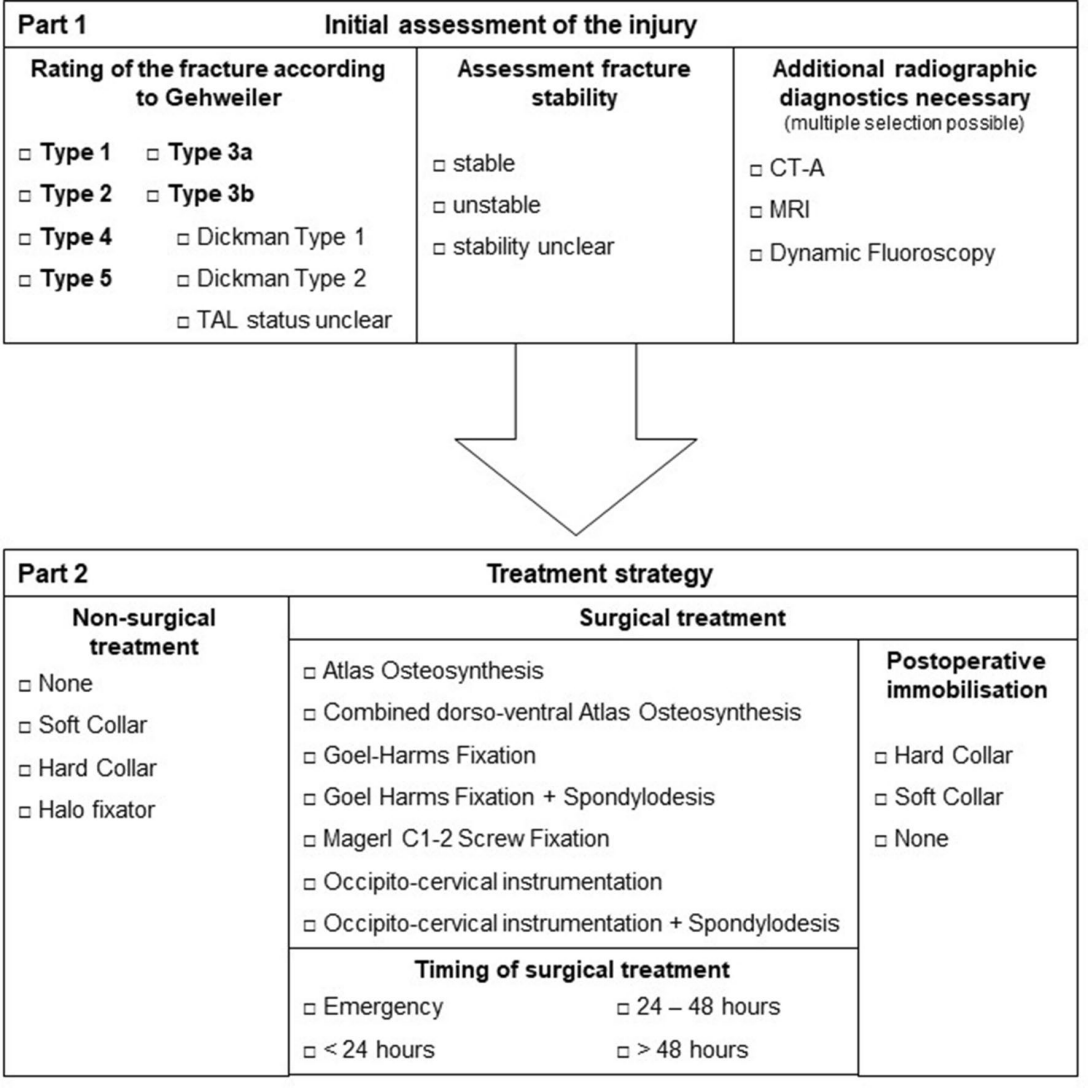
Anonymised questionnaire with initial assessment of the atlas fracture (part 1) and suggestions for treatment strategy (part 2). *CT-A* computed tomography angiography, *MRI* magnetic resonance imaging, *TAL* transverse atlantal ligament

Each observer reviewed all 34 cases. The website provided a plain, anonymous radiograph image and computed tomography (CT) sequences in axial, coronal, and sagittal reconstructions of isolated atlas fractures (Fig. 2) as well as case-specific information (age, sex, neurological status, and injury mechanism). In addition, each observer was provided with an overview of the Gehweiler classification. This included detailed information about its five subgroups and the subdivision of type 3 fractures into types 3a and 3b as well as the classification of TAL lesions according to Dickman et al. [15]. Within the first part of the questionnaire (Fig. 3, part 1), the observers independently rated the fracture according to the Gehweiler classification, and in the case of a type 3b fracture, the integrity of the TAL was assessed by applying the Dickman classification. Furthermore, the observers were asked to rate the overall stability of the fracture (stable, unstable, or stability unclear) and the need for additional radiographic diagnostics. Within the second part of the questionnaire (Fig. 3, part 2), the observers were asked to recommend either surgical or non-surgical treatment and to provide further treatment suggestions.

### Data analysis

All analyses of anonymised data were conducted using SPSS version 25 (IBM). Interobserver reliability was calculated using Fleiss’ kappa (*κ*) statistics [16] with the critical value for significance set to *p* < 0.05. No reference standard (“gold standard”) for grading the C1-fractures according to the Gehweiler classification or the treatment choices against which to compare observer’s recording was considered during this analysis. To determine the degree of agreement, interobserver reliability was graded as described by Landis and Koch [17], with a value of 1.00 indicating perfect reliability, 1.00–0.81 nearly perfect, 0.80–0.61 substantial, 0.60–0.41 moderate, 0.40–0.21 fair, and ≤ 0.20 poor reliability.

## Results

Cases rated by the six board-certified spine surgeons had a mean age of 64.0 years (± 17.7), 52.9% were men, and none had pathologic neurological findings according to the American Spinal Cord Injury Association Impairment Scale (ASIA E). Most often (64.8%) the origin of the injury was a fall, either from the same (32.4%) or an elevated (32.4%) level (Table 1). In total, 204 case-specific ratings of individual atlas fractures according to the Gehweiler classification (Table 2) and consecutive treatment strategies were obtained.

**Table 1:** Demographic data of cases

Characteristics	Number (%)
Age—years, mean (± SD)	64.0 (± 17.7)
Sex	
Male	18 (52.9%)
Female	16 (47.1%)
Neurological status	
ASIA E (normal sensation and motor function)	34 (100%)
Injury mechanism	
Fall—same level	11 (32.4%)
Fall—elevated level	11 (32.4%)
Traffic accident	7 (20.6%)
Other	5 (14.6%)

*ASIA* American Spinal Cord Injury Association Impairment Scale, *SD* standard deviation

**Table 2:** Interobserver reliability of the Gehweiler classification

Gehweiler classification	*N*	Kappa value (*κ*)
Type 1	19	0.52
Type 2	7	0.85
Type 3a	20	0.31
Type 3b	90	0.53
Type 4	66	0.53
Type 5	2	− 0.10
Overall	204	0.50

In total 34 cases of isolated atlas fractures were rated by six observers. Therefore, in total 204 case-specific ratings of individual atlas fractures (*N* = 204) according to the Gehweiler classification are represented

### Interobserver reliability

For the Gehweiler classification we observed a moderate interobserver reliability (*κ* = 0.50, *p* < 0.0005). The Fleiss’ kappa statistics for the Gehweiler types 1–4 proved to be statistically significant (all *p* < 0.0005). While for the estimated kappa value (*κ* = − 0.01) of the type 5 fracture (*N* = 2), its calculation due to chance could not be ruled out (*p* = 0.823). The highest interobserver reliability coefficient is observed for the fracture type 2 (*κ* = 0.85) whereas the fracture type 3a (*κ* = 0.31) has the lowest kappa value (Table 2). When including the differentiation of TAL integrity in Gehweiler type 3b fractures (Dickman type 1 or type 2 or TAL status unclear) Fleiss’ kappa was 0.39.

The data analysis indicated that 11 of the 34 cases were unanimously rated by the observers as Gehweiler type 3 fractures. In the subsequent statistical analysis of these eleven cases an estimated kappa value of 0.35 (*p* < 0.0005) was observed for the distinction between type 3a and type 3b fractures and an estimated kappa value of 0.28 (*p* < 0.0005) for the assessment of stability.

Overall, for the stability assessment of isolated atlas fractures, the kappa value was 0.50, and for the decision regarding surgical or non-surgical therapy, the kappa value was 0.53.

### Stability assessment, additional radiographic diagnostics, and treatment regime

In the first part of the questionnaire, the observers rated atlas fractures according to the Gehweiler classification, assessed the fracture stability, and suggested either a surgical or non-surgical treatment regime (Table 3). Additionally, as part of the initial fracture assessment in individual cases, the observers could make suggestions for further radiographic diagnostics they deemed necessary. Several additional radiographic diagnostics were possible for each individual case. A detailed presentation of additional radiological diagnostics recommended by the observers is shown in Table 4.

**Table 3:** Stability assessment and treatment regime of atlas fractures

Gehweiler classification	*N*	Stability (in %)			Treatment (in %)	
	204	Stable	Unstable	Stability unclear	Surgical therapy	Non-surgical therapy
Type 1	19	94.7	–	5.3	–	100.0
Type 2	7	100.0	–	–	–	100.0
Type 3a	20	65.0	–	35.0	5.0	95.0
Type 3b	90	4.4	86.7	8.9	90.0	10.0
Type 4	66	60.6	25.8	13.6	25.8	74.2
Type 5	2	100.0	–	–	–	100.0

**Table 4:** Suggested additional radiographic diagnostics

Gehweiler classification	*N*	Additional radiographic diagnostic				
		CT-A (%)	MRI (%)	Dynamic fluoroscopy (%)	No further imaging necessary (%)	No information supplied (%)
Type 1	19	31.6	42.1	10.5	31.6	5.3
Type 2	7	42.9	57.1	–	42.9	–
Type 3a	20	20.0	75.0	15.0	15.0	5.0
Type 3b	90	68.9	43.3	8.9	5.6	2.2
Dickman 1	7	42.9	85.7	–	–	–
Dickman 2	57	75.4	21.1	3.5	8.7	3.5
TAL status unclear	26	61.5	80.8	23.1	–	–
Type 4	66	68.2	40.9	6.1	13.6	3.0
Type 5	2	100.0	–	–	–	–

It was possible to suggest multiple additional radiographic diagnostics in each case. *CT-A* computed tomography angiography, *MRI* magnetic resonance imaging, *TAL* transverse atlantal ligament

#### Gehweiler type 1

Type 1 fractures (*N* = 19) were rated as stable in 94.7% of cases. Non-surgical therapy was recommended for all cases. In 31.6% of type 1 fractures, additional computed tomography angiography (CT-A) was recommended. In 42.1%, additional magnetic resonance imaging (MRI) was recommended.

#### Gehweiler type 2

Type 2 fractures (*N* = 7) were rated as stable in 100% of cases, and 100% were treated non-surgically. Of type 2 fractures, additional CT-A was recommended in 42.9% and MRI in 57.1% of cases.

#### Gehweiler type 3a and type 3b

Type 3a fractures (*N* = 20) were rated as stable in 65.0% of cases, while stability was unclear in 35.0% of cases. Surgical treatment was recommended in 5.0% of cases rated as Gehweiler type 3a fractures. For further assessment of type 3a fractures, 20.0% of the observers suggested an additional CT-A, and 75.0% recommended performing an MRI.

Type 3b fractures (*N* = 90) were assessed as unstable in 86.7% of cases, and surgical treatment was recommended in 90.0%. For type 3b fractures, additional imaging tools recommended were CT-A (68.9%) and MRI (43.3%). The subdivision of Gehweiler type 3b fractures regarding TAL integrity showed that in lesions rated as Dickman type 1, additional CT-A (42.9%) and additional MRI (85.7%) were recommended. Additional CT-A was recommended in 75.4% of cases with TAL lesions specified as Dickman type 2, and additional MRI was recommended in 21.1% of these cases. In cases in which TAL integrity was unclear, especially additional MRI (80.8%) was recommended.

#### Gehweiler type 4

In total, 60.6% of type 4 fractures (*N* = 66) were rated as stable, and surgical treatment was suggested in 25.8% of these cases. In type 4 fractures, 68.2% of the observers suggested additional CT-A, and 40.9% of raters suggested additional MRI.

#### Gehweiler type 5

C1 fractures were rarely graded as type 5 fracture (N = 2). Both type 5 fractures were rated as stable and non-surgical therapy was recommended. In each type 5 fracture case, the observers suggested additional CT-A.

### Timing of surgical treatment

The observers rated the urgency of operative treatment for each atlas fracture and did not recommend emergency surgery in any case. The suggested timeframe for the surgical treatment of Gehweiler type 3a fractures in this study was 24–48 h after diagnosis. The timeframe of surgery for Gehweiler type 3b fractures was within 24‒48 h (37.0%) or later than 48 h (43.2%). For type 4 fractures, the observers recommended surgical treatment somewhat more promptly, with 70.6% being recommended for treatment within the first 48 h.

### Surgical technique

Surgical treatment was mainly recommended for type 3b (in 90% of cases) and type 4 (in 25.8% of cases) fractures. It was only recommended in one of 20 type 3a cases, which involved atlas osteosynthesis (Table 5). In Gehweiler type 3b fractures, isolated C1 fixation by posterior stabilisation was most frequently (65.4%) recommended followed by occipito-cervical instrumentation (23.5%). Additional fusion was recommended in 73.6% of those patients in whom an indication for an occipito-cervical instrumentation was seen.

**Table 5:** Suggested techniques for surgically treated atlas fractures

Gehweiler classification	Surgical/non-surgical treatment (*N*)	Atlas osteo-synthesis (%)	Combined dorso-ventral atlas osteo-synthesis (%)	Goel–Harms fixation (%)	Magerl C1–2 screw fixation (%)	Occipito-cervical instrumentation (%)
Type 3a	1/20	100	–	–	–	–
Type 3b	81/90	65.4	1.2	8.6	1.2	23.5
Dickman 1	7/7	42.9	–	28.6	–	28.6
Dickman 2	55/57	66.7	–	8.8	1.8	19.3
TAL status unclear	19/26	46.2	3.8	–	–	23.0
Type 4	17/66	5.8	–	–	–	94.2

*TAL* transverse atlantal ligament

Detailed analysis of Gehweiler type 3b fractures according to TAL integrity showed that the recommended treatment options for Dickman type 1 lesions were atlas osteosynthesis (42.9%), Goel–Harms fixation (28.6%), and occipito-cervical instrumentation (28.6%). For Dickman type 2 lesions, suggested treatment options were atlas osteosynthesis (66.7%) and occipito-cervical instrumentation (19.3%). Atlas osteosynthesis was recommended in 46.2% of cases in which TAL status was rated unclear, and occipito-cervical instrumentation was recommended in 23.0%. The suggested surgical treatment for Gehweiler type 4 fractures was mainly occipito-cervical instrumentation (94.2%). Additional spondylodesis was recommended in 43.8% of cases in which occipito-cervical instrumentation was recommended (Table 5).

### Cervical immobilisation

Non-surgical treatment using a cervical collar was recommended for all Gehweiler type 1, 2, and 5 fractures and for 95% of Gehweiler type 3a fractures. A cervical collar was often recommended for the postoperative protection of the cervical spine. For example, this was recommended in 74.5% of isolated dorsal C1 osteosyntheses and 88.6% of cases of occipito-cervical instrumentations. After isolated dorsal atlas osteosynthesis, one-quarter (25.5%) of the observers reported no need for additional post-operative protection.

## Discussion

Reliable fracture classification systems are crucial, as they form the basis for treatment algorithms and help clinicians understand and communicate injury pathomechanism. Our main findings were as follows:

1. We found a moderate interobserver reliability for the Gehweiler classification while the lowest kappa value was observed for the fracture type 3a.
2. We further observed a moderate interobserver reliability for C1-fracture stability assessment. While stability assessment and treatment recommendations in cases rated as Gehweiler type 3a, type 3b or type 4 vary considerably among observers, spine surgeons highly agreed that C1-fractures graded as Gehweiler type 1, type 2 or type 5 may be considered as stable and treated non-surgically.
3. Furthermore, we found a moderate interobserver reliability regarding the decision to recommend surgical or non-surgical treatment, which was independent of the initial fracture classification. We observed in particular variation in the suggested treatment of Gehweiler type 3b fractures.

Previous studies on the reliability of spine fracture classification systems have reported inferior interobserver results for the Thoracolumbar Injury Classification and Severity Scale (*κ* = 0.23–0.29) [18, 19]. However, studies have shown slightly superior interobserver reliability for the AOSpine Thoracolumbar Spine Injury Classification System (*κ* = 0.55–0.59) [18, 20, 21], and the AOSpine Subaxial Cervical Injury Classification (*κ* = 0.56) [22]. In a small sub-sample of a study investigating the interobserver reliability of the Swedish Fracture Register for Vertebral Fractures, atlas fractures (*N* = 8) were rated according to the Jackson classification [23] by four orthopaedic surgeons and one student. Substantial interobserver reliability was reported (*κ* = 0.785) [4]. However, the interpretation and generalizability of findings from this study were limited due to the small number and homogenous morphology of atlas fractures, which consisted of two posterior arch fractures, five burst fractures, and one lateral mass fracture. Therefore, by applying the Gehweiler classification, we demonstrate, for the first time, a statistically valid interobserver reliability for the classification of isolated atlas fractures.

Characteristics of the 34 cases in the present study appropriately reflected the gender distribution, mean age, and injury mechanism of patients suffering from isolated atlas fractures [2, 6] as well as the fact that neurological deficits are very rarely observed [24, 25]. Pathological neurological findings were not observed in any cases, so in line with the literature, surgical intervention with early decompression was not indicated, and non-emergency surgery was justified [26]. However, moderate interobserver reliability for the evaluation of fracture stability and the need for surgical or non-surgical treatment, which was assessed independent of the Gehweiler rating, suggested a relevant discrepancy in the assessment of fracture stability. Among the observers, there was a strong consensus on treatment strategy for Gehweiler type 1, 2, 3a, 4, and 5 fractures; however, a lack of consensus was observed for Gehweiler type 3b fractures. These challenges were in line with the reported highly demanding clinical and biomechanical assessment of isolated C1 fracture stability [27, 28].

Except for one case, Gehweiler type 1, 2, and 5 fractures were rated as stable, and non-surgical treatment with a cervical collar was recommended for these cases. Using this conservative treatment as the method of choice was in line with recommendations for the treatment of atlas fractures of the spine section of the German Society for Orthopedics and Trauma [10]. In line with recommendations for the diagnostic testing of Gehweiler type 5 atlas fractures, additional CT-A was required due to their anatomical vicinity to the vertebral artery [11]. Interestingly, although the vast majority of the observers rated Gehweiler type 1 and 2 fractures as stable and recommended non-surgical therapy, they recommended additional radiographic diagnostics (CT-A and MRI) in half the cases. For economic reasons and in view of the limited availability of radiological resources, in the opinion of the authors, the indication for additional imaging in Gehweiler type 1 and 2 fractures should be critically questioned.

In cases rated as Gehweiler type 3a fractures, 35.0% of the observers could not assess stability and the lowest interobserver reliability of all types of atlas fractures is observed. However, non-surgical treatment was chosen in all cases except one. An intact TAL is part of the definition of a Gehweiler type 3a fracture [14]. Due to the stability inherent in cases with an intact TAL, non-surgical treatment of this type of fracture is widely accepted. Nevertheless, careful follow-up is necessary to identify further dislocation, non-union, and/or signals of atlantoaxial instability as early as possible [29]. In 75.0% of cases rated as Gehweiler type 3a fractures, additional MRI was ordered, reflecting the need for practitioners to visualise this type of fracture using imaging that is capable of analysing the ligamentous status of the craniocervical junction and to rule out hidden type 3b fractures after an upper cervical spine injury [30].

For Gehweiler type 3b fractures, additional CT-A was recommended in more than two-thirds of cases and is most likely to be interpreted in the context of preoperative preparation. Due to the heterogeneous shape of the C1 posterior arch and the vertebral artery, preoperative CT-A is crucial to identify anatomical variations, such as an anomalous vertebral artery, and reduce the risk of intraoperative vertebral artery injury [31] and difficulties with screw placement [32]. Gehweiler type 3b fractures were rated unstable in 86.7% of cases, and surgery was recommended in 90%. Isolated C1 fixation using posterior stabilisation was most frequently recommended. In comparison to atlantoaxial or occipito-cervical fusion, which was the treatment methods of choice for unstable atlas fractures for many decades, atlas osteosynthesis provides inherent stabilisation limited to the atlas, which may preserve motion [29]. Consequently, isolated atlas osteosynthesis may be associated with shorter surgery durations, lessened surgical trauma, and fewer complications.

In cases in which the observers assumed an intraligamentous lesion (Dickman type 1) or when the status of the TAL was unclear, additional MRI was requested. In addition, in these cases, the observers suggested the fixation of multiple segments (for example, C1–C2 or C0–C2) rather than an isolated C1 posterior fixation more often than in the surgical treatment of type 3b fractures, which had bony avulsions of the TAL. This was in line with the assumptions that an intraligamentous lesion of the TAL results in translational atlantoaxial instability and that these Dickman type 1 lesions require C1‒2 fusion [15]. However, in line with the fact that this paradigm has been increasingly questioned, nearly half of our cases of Dickman type 1 rupture and those with unclear TAL status were treated using isolated atlas osteosynthesis. The rationale for this surgical treatment may be related to the fact that isolated osteosynthesis of the atlas in cases of violated TAL integrity was not associated with atlantoaxial stability on flexion/extension films in Dickman type 1 and type 2 injuries. This was reported by Shatsky et al. [28] in their retrospective case-controlled study (*N* = 12). Historically, the necessity of C1‒2 fixation for intraligamentous TAL lesions originated from ligamentous translational atlantoaxial instability of affected TALs in patients suffering from rheumatoid arthritis who had rheumatoid processes involving multiple ligaments of the upper cervical spine. Isolated atlas fractures result from traumatic compression injuries and, therefore, are commonly associated with intact secondary stabilisers, including further ligaments of the upper cervical spine. In addition, Koller et al. [27] reported in their biomechanical investigation of displaced Jefferson burst fractures appropriate C1‒2 stability by conducting an isolated atlas osteosynthesis.

Nearly two-thirds of Gehweiler type 4 fractures were rated stable, and surgery was favoured in one-quarter of the cases, while occipito-cervical instrumentation was suggested for all cases except two. Thus, surgical treatment was recommended for Gehweiler type 4 fractures rated as unstable. The instability of type 4 fractures may occur as a result of a fractured lateral mass that is primarily or secondarily significantly dislocated or in cases of a sagittal split fracture of the lateral mass [33]. In those cases, the treatment guidelines of the German Society for Orthopaedics and Trauma recommend halo traction for 6‒12 weeks or temporary occipito-cervical stabilisation without fusion to avoid the development of persistent pain caused by post-traumatic arthritis [10]. However, especially in the elderly, problems caused by the invasiveness of this conservative form of halo traction might outweigh potential surgical complications [34, 35]. During upper cervical trauma, many vascular structures are at risk, especially the vertebral artery, which is also endangered in Gehweiler type 4 lesions when the foramen of the transverse process is involved. Therefore, in line with the recommendation for additional CT-A to exclude lesions of the vertebral artery [36], the observers in this study called for CT-A in 68.2% of Gehweiler type 4 fractures. Blunt injury to the vertebral artery may result in a stroke, a loss of posterior circulation to the brain, and neurologic sequelae that have potentially devastating consequences [37].

Postoperative cervical immobilisation using a soft or hard collar was recommended in most cases, and the rationale for this was most likely the well-known reduction in pain and spinal stability it provides [38, 39]. However, in particular the indication for postoperative immobilisation in cases of surgical treatment by occipito-cervical instrumentations should be critically questioned due to the appropriate stabilisation and minor residual range of motion that is inherent with this surgery. Therefore, in cases of occipito-cervical instrumentations, the risk of complications associated with postoperative immobilisation may outweigh the benefit of such an immobilisation.

The present study had some limitations. First, it can be argued that a sample size of 34 isolated atlas fractures is too small to provide an appropriate understanding of interobserver reliability. This could be the focus for future studies. However, finding a larger number of isolated atlas fractures would be difficult, and the requirement to evaluate a larger sample size might negatively influence the response rate of experienced spine surgeons. Additionally, the small number of the observers may limit our reliability assessment [40]. However, experienced spine surgeons from Level-I Trauma Centers guide the management of atlas fractures, and the role of training on the size and precision of reliability estimates is crucial [41]. Furthermore, the Gehweiler classification is commonly applied in Europe, but in North America and Asia, the Jefferson [42] classification is widely used. Therefore, the generalizability of interobserver agreement might differ depending on the working location of individual spine surgeons. In contrast to the Jefferson classification, the Gehweiler classification differs in the mandatory distinction between type 3 fractures as stable (3a) or unstable (3b). This distinction in particular forms the basis for the decision for a non-surgical or surgical therapy. In the opinion of the authors, the use of the Gehweiler classification is therefore especially beneficial in the assessment and treatment of atlas fractures. Evaluating the integrity of the TAL, and thus the possibility of distinguishing a stable type 3a and an unstable type 3b fracture, might have been easier with the additional radiographic information provided by an MRI scan. This assumption is supported by our findings of only fair interobserver reliability for the subtype distinction between type 3a or type 3b fractures (*κ* = 0.35) (Fig. 2 illustrates an exemplary case). Further on, in these cases stability assessment is even more impaired (*κ* = 0.28). Therefore, a higher interobserver reliability might have been achieved with the availability of MRI scans. However, testing the reliability of a global classification system should not rely on an imaging tool that has limited availability. For this reason, we did not provide MRI scans of the cases. Nonetheless, since the treatment strategy is decisively guided by the distinction between type 3a and type 3b fractures, the performance of MRI diagnostics is mandatory in the case of Gehweiler type 3 fractures [13].

## Conclusion

The Gehweiler classification and stability assessments of isolated atlas fractures had moderate interobserver reliability in this study of experienced spine surgeons. For stability assessment and treatment recommendation high level of interobserver agreement was observed for C1-fractures rated as Gehweiler type 1, type 2 and type 5 but consensus was considerably less for type 3a, type 3b and type 4 fractures. Therefore, further biomechanical and clinical investigations are necessary to broaden our understanding of atlas fractures and refine treatment guidelines that integrate the therapeutic and prognostic implications of the Gehweiler classification system. Future studies may also want to determine whether atlas osteosynthesis in unstable C1-fractures with intraligamentous rupture or displaced bony avulsion of the TAL is reliably associated with atlantoaxial stability and good outcomes.

## Data Availability

The datasets used during the current study are available from the corresponding author on reasonable request.
